# Comparative analysis of genomic characteristics, virulence and fitness of community-associated *Staphylococcus aureus* ST121 clone causing fatal diseases in China and other CA-MRSA clones

**DOI:** 10.1080/21505594.2023.2242547

**Published:** 2023-08-03

**Authors:** Wangxiao Zhou, Ye Jin, Gaoqin Teng, Weiwei Chen, Yunbo Chen, Qixia Luo, Yonghong Xiao

**Affiliations:** a State Key Laboratory for Diagnosis and Treatment of Infectious Diseases, National Clinical Research Center for Infectious Diseases, Collaborative Innovation Center for Diagnosis and Treatment of Infectious Diseases, the First Affiliated Hospital, Zhejiang University School of Medicine, Hangzhou, People’s Republic of China; b Department of General Intensive Care Unit, The Second Affiliated Hospital of Zhejiang University School of Medicine, Hangzhou, Zhejiang, People’s Republic of China; c Department of Laboratory Medicine, the First Affiliated Hospital, School of Medicine, Zhejiang University, Hangzhou, People’s Republic of China; d Department of Structure and Morphology, Jinan Microecological Biomedicine Shandong Laboratory, Jinan, People’s Republic of China; e Research Units of Infectious Disease and Microecology, Chinese Academy of Medical Sciences, Beijing, China

**Keywords:** ST121, community-associated infections, genomic analysis, high virulence, epidemiological success

## Abstract

The increasing rate of community-associated *Staphylococcus aureus* (CA-SA) worldwide has aroused global public concern for decades. Although ST121 clone is one of the prevalent CA-SA in China, there is still limited knowledge about it. In this study, we conducted a genomic analysis of 28 CA-SA ST121 isolates from severe bloodstream infection cases and 175 ST121 isolates from the public database. Phylogenetic analysis revealed the consistency and the complexity of global ST121 lineages, and suggested potential cross-country even cross-continental transmission of ST121 isolates. By investigating the virulence and fitness between ST121-CA-methicillin-resistant SA (CA-MRSA) and other CA-MRSA clones, we found that ST121-MRSA exhibits virulence comparable to the highly virulent USA300 clone, exceeding that of the predominant CA-MRSA lineage ST59 in China and the other American CA-MRSA clone MW2. Notably, based on analyses of virulence genes, *eta*, *etb*, *edin-C* and egc were only found in ST121, suggesting that the high virulence of ST121 may be attributed to the combination of these virulence factors encoded by mobile genetic elements. However, results of experiments in mice nasal and human alveolar epithelial cells showed that the colonization capacity of ST121 is much lower than that of other clones. Moreover, ST121-MRSA displayed much lower acid tolerance, suggesting that ST121-MRSA may not have such capacity to achieve the epidemiological success of other CA-MRSA clones and become the dominant lineage. Our findings expand current understanding of the epidemiology and pathogenicity of the hypervirulent ST121 clone, and highlight the importance of colonization capacity and environmental adaption in MRSA epidemiological success.

## Introduction

*Staphylococcus aureus* is one of the most successful pathogens and capable of causing infections ranging from minor skin and soft tissue infections to fatal conditions [1]. When *S. aureus* infects the host, a variety of virulence factors can modulate the immune responses of the host to ensure bacterial survival. However, various clonal lineages appear to have different potential to cause severe disease, which is generally attributed to the presence or absence of particular virulence factors or to the levels at which these virulence factors are expressed [2,3]. Community-associated methicillin-resistant *S. aureus* (CA-MRSA) is generally considered to be more virulent and produces a greater amount of toxins compared to hospital-associated MRSA. As a result, CA-MRSA clones tend to cause both acute and severe diseases [4].

As one of the prevalent CA-SA clones in China [5,6], ST121 clone often causes both superficial skin infections and severe invasive infections such as neonatal skin blistering disorders, wound infections, and sepsis [6–8]. A previous study investigated 201 *S. aureus* ST121 isolates obtained from 11 published studies and found that almost all (92.0%, 185/201) harboured Panton Valentine leucocidin (PVL) [8], which is a pore-forming protein exotoxin that plays important roles in antagonizing the host defence against *Staphylococcus* and triggers apoptosis and lysis of infected cells. In addition, some ST121 strains can produce exfoliative toxins (ETs) ETA and ETB that can cause staphylococcal scalded skin syndrome (SSSS) and bullous impetigo. ST121 is globally distributed, and this clone is generally susceptible to methicillin. However, previous studies have underestimated the importance of this hypervirulent methicillin-susceptible *S. aureus* (MSSA) clone.

Similar to certain CA-SA lineages including USA300 (ST8) in the USA, ST72 in South Korea and ST59 in China that have emerged as potential causes of severe nosocomial infections [9–11], in this study, we isolated and sequenced the genomes of 28 ST121 CA-SA isolates including 6 MRSA isolates and 22 MSSA isolates, which caused fatal infectious diseases. A phylogenetic analysis was conducted to investigate the genetic features and evolutionary history of global ST121 clone. Furthermore, a comparative analysis of virulence and fitness between representative global CA-MRSA clones (ST59, ST8 and ST1) and ST121 clone was performed to better understand the differences between these dominant CA-MRSA lineages and the globally disseminated hypervirulent clone.

## Methods and materials

### Collection of bacterial isolates

A total of 3,569 *S. aureus* isolates were collected from patients with bloodstream infections (BSIs) in 18 provinces of China from 2010 to 2019. Identification and multi-locus sequence typing of the *S. aureus* isolates was performed by MALDI-TOF/MS and sequencing of seven house-keeping genes (*gmk*, *glpF*, *arcC*, *aroE*, *tpi*, *yqiL*, and *pta*). Finally, based on the criteria for CA *S. aureus* [6], 28 ST121 CA-SA isolates (6 MRSA and 22 MSSA) were identified and subjected to whole-genome sequencing.

### Whole-genome sequencing and genomic analysis of ST121 isolates

Whole-genome sequencing of ST121 isolates was carried out using a HiSeq X Ten platform (Illumina, San Diego, CA, USA) with a 2 × 150 bp read length. SPAdes v3.14.1 [12] was used to assemble the clean reads obtained after adaptor trimming and quality filtering (Phred quality score ≥ 20) by fastp v0.20.1 [13]. The web-based SCCmecFinder (https://cge.cbs.dtu.dk/services/SCCmecFinder) and SpaFinder (https://cge.cbs.dtu.dk/services/spatyper) programs were used to confirm the SCC*mec* and *spa* typing of ST121 isolates, respectively. Additional 175 ST121 genomic assemblies with geographic information were downloaded from the NCBI GenBank database for comparison (**Table S1**).

### Phylogenetic analysis and comparison of virulence factors, pathogenicity islands, and prophages among ST121 isolates

We used Snippy v4.6.0 (https://github.com/tseemann/snippy) to identify single nucleotide polymorphisms (SNPs) in the ST121 core genome, with the *S. aureus* XQ genome (ST121, GenBank accession no. CP013137.1) used as the reference for read mapping. SNPs falling within the recombined regions of the core genome detected using Gubbins v2.4.1 were excluded from the core genome, and a phylogenetic tree for ST121 isolates was constructed using the core SNPs with RAxML [14] (1000 bootstrap replications). The output from Gubbins was loaded directly to BactDating v1.1 [15] for generating a time-resolved tree of ST121 isolates. We used RASP v4.2 [16], which implements Bayesian binary MCMC method to estimate the ancestral origins of ST121 isolates with 10 different chains during 50 million generations. Minimum spanning trees (MSTs) based on core SNP data were constructed using PHYLOViZ v2.0 (https://online.phyloviz.net/index). Virulence factors, prophages and genomic islands were identified using VFanalyzer (http://www.mgc.ac.cn/cgi-bin/VFs/v5/main.cgi?func=VFanalyzer), PHASTER [17] and IslandViewer 4 [18], respectively.

### Comparison of survival rates of *Galleria mellonella* larvae infected with different CA-MRSA clones

Overnight cultures were harvested by centrifugation (6000 rpm, 10 min), washed thrice with sterile PBS, then resuspended in PBS (pH 7.2) and adjusted to a concentration of 1 × 10^6^ CFU/mL. Larvae (*n* = 20) were injected with 20 μL of the respective strains. Negative control was injected with 20 μL sterile PBS. The larvae were then placed in a clean, sterile plastic petri dish and cultured at 37°C. The number of dead larvae was recorded within 72 h.

## Mouse model of skin infection assay

Eight-week-old, female, BALB/C-nude mice were used for the study. Each mouse was housed for 1 week prior to infected with *S. aureus*. An overnight bacterial culture was harvested by centrifugation (6000 rpm, 10 min), washed thrice with sterile PBS and suspended in sterile PBS (pH 7.2) at a concentration of 1 × 10^8^ CFU. Each mouse was given a hypodermic injection with equivalent *S. aureus* suspension (100 μL), normal food and drinks. The normal blank group was infected with sterile PBS and used as negative control. The size of skin abscesses was recorded as length and width using a caliper. The abscess area was calculated by the following equation: abscess area=length (L)×width (W).

## Biofilm formation assay

Isolates were incubated in a 96-well microtiter plate containing 0.5% glucose in 200 μL TSB medium at 37°C. After 24 h of incubation, the microplate was washed with PBS, and the biofilm adhered to the microwells was fixed with 95% methanol. After drying, the biofilms in every well in the plate were stained with 1% crystal violet for 10 min. Next, 33% glacial acetic acid was added to dissolve the biofilm, which was quantified by recording the OD_600_ of each well.

## Mice nasal colonization model

A total of 10 μL of sterile PBS containing bacterial cells (at a concentration of 0.5 McFarland turbidity standards) was dropped into the nasal cavity of each mouse (*n* = 5). After 5 days housed, the mice were euthanised, and the nose was dissected and homogenised. Total *S. aureus* counts were determined by plating 200 μL diluted tissue homogenate onto TSA petri dish.

## Adhesion assay

The adhesion capacity of different clones was evaluated as previously described [19]. Overnight cultures were diluted to an OD_600_ value of 0.5. Next, A549 human alveolar epithelial cells and bacterial cells were co-incubated in a 37°C incubator containing 5% CO2. After 2 h of incubation, floating bacterial cells were removed, and A549 cells were dissolved by addition of 0.1% deoxysodium cholate solution.

## Growth at acid pH

TSB medium was adjusted to pH 5.5 using hydrochloric acid. OD_600_ values were measured hourly for the first 6 h, and at 16 h and 24 h following inoculation [20].

## Statistical analysis

All assays were repeated three times independently. Kaplan – Meier analysis was used to assess differences in the survival rates of *G. mellonella* larvae infected with different CA-MRSA clones. Wilcoxon rank-sum test was used for comparing skin lesions between mice and results of nasal colonization and adhesion assay. All data were analysed using GraphPad Prism 9.0 software.

## Data availability

The whole-genome sequences of 28 ST121 *S. aureus* isolates determined in this study have been submitted to the DDBJ/EMBL/GenBank database under BioProject accession no. PRJNA787011.

## Results

### Brief clinical reports of CA-SA ST121 strains

Twenty-eight CA-SA ST121 strains were collected from severe BSIs in different patients in China between 2010 and 2019, including 10 children and 18 adults. The median age of patients was 34 years (30.34 ± 24.92 years; range: two weeks to 72 years), and 12 patients (42.86%) were males. There were 19 cases of primary skin and soft-tissue infection (8 children with SSSS) and 9 cases of pneumonia. Six patients ultimately died of sepsis. The clinical information of these patients is presented in Table S2.

## Geographic distribution and phylogenetic analysis of global *S. aureus* ST121 clone

**Figure 1.**
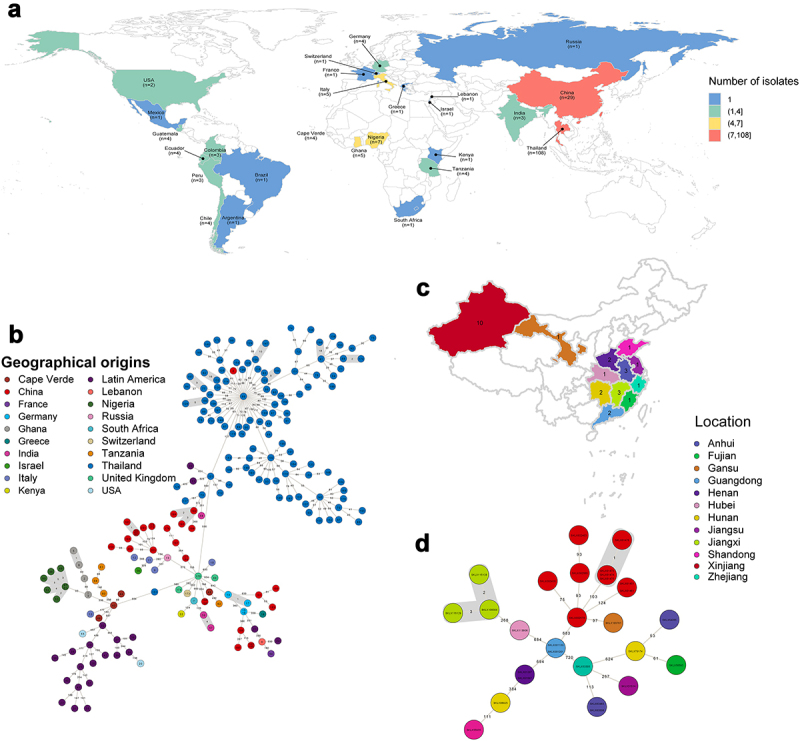
The geographical distribution and genetic relationships among 203 ST121 isolates examined in this study. (a & c) Geographical distribution of 203 ST121 *S. aureus* isolates around the world and 28 ST121 from our collection in China. The countries or Chinese provinces with ST121 isolates are marked by background colour fill. (b & d) MST of 203 ST121 *S. aureus* isolates around the world and 28 ST121 from our collection in China. Each filled circle represents a ST121 isolate. The number of SNPs in pairwise comparisons is indicated on the connecting lines. The potential transmission clusters based on the threshold of 23 SNPs are marked with grey.

To investigate the molecular evolution of ST121 strains in this study, 28 ST121 *S. aureus* genomes from our collection and additional 175 publicly available ST121 genomes sampled between 1932–2020 were analysed. The geographic distribution of ST121 is shown in (Figure 1a), including 27 countries, such as Thailand (*n* = 108), China (*n* = 29), Nigeria (*n* = 7), Germany (*n* = 4) and the USA (*n* = 2), covering samples from Asia, Europe, America and Africa. The 28 ST121 *S. aureus* strains from China were isolated across 12 provinces from patients with severe and fatal community-associated infections (Figure 1c), with the highest number of strains from Xinjiang Province (*n* = 10). The remaining isolates from China in our collection were obtained from 11 provinces within close geographic proximity, suggesting potential interregional dissemination of ST121 isolates in China (Figure 1c). Detailed information regarding these ST121 strains is shown in **Table S1**.

**Figure 2.**
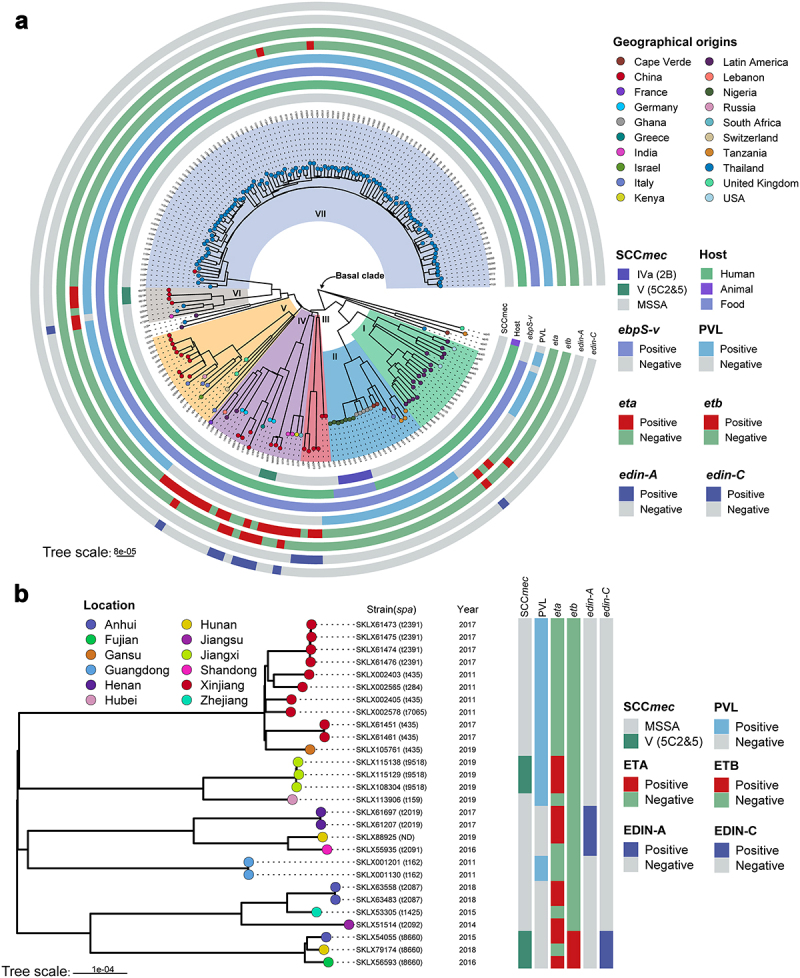
Phylogenetic trees of ST121 isolates. (a) Phylogenetic tree of 203 ST121 isolates from around the world. Tips are labelled by *spa* type of the isolates and coloured according to country of isolation. The SCC*mec* type, host type, presence of *ebpS-v*, PVL, *eta*, *etb*, *edin-A*, and *edin-C* are mapped on the tree (from inner to outer circle). (b) Phylogenetic tree of 28 ST121 isolates from our collection in China. Tips are coloured based on provinces in China.

All ST121 strains were divided into seven phylogenetic clades (Figure 2a), with a common ancestor dated to ~ 1770.87 (95% highest posterior density [HPD] interval, 1713.88 to 1814.05) (Figure S1). Strains of clade I were all obtained from America (North America and Latin America), and the main *spa* type was t645 (57.89%, 11/19). In clade II, all ST121 strains belonged to unique *spa* type t314. Within clade VII, almost all ST121 strains were isolated from Thailand, except one Chinese isolate, XQ. Furthermore, Chinese ST121 clones were distributed in clades III, IV, V, VI, and VII. Notably, within clade IV, the ST121 MRSA isolates belonging to ST121-V-t8660 were PVL-negative clones, whereas in clade VI, the ST121-V-t9518 clones were PVL positive, and the remaining isolates were all MSSA. These results suggest that the Chinese ST121 MRSA clones comprise two distinct lineages with different evolutionary origins, possibly emerging in ~ 2002.66 (ST121-V-t8660) and ~ 2017.18 (ST121-V-t9518), respectively (Figure S1). In addition, although there were some clusters of closely related strains from the same countries, we found that strains from different countries or different provinces in China clustered (Figure 1b,d) suggesting that cross-country transmission and interregional transmission within China may occur.

Notably, in our reconstructions of the geographic origins for ST121 clones, we found that the ancestors of ST121 isolates from Asia have a predicted origin in China, with posterior probability of 95.5% (Figure S2). These results suggest the extant Asian ST121 isolates might trace to transmission events from China followed by local diversification, such as local extension of ST121 clones from Thailand (Figure 2a).

### Virulence genes and mobile genetic elements (MGEs) in ST121 and comparison with other CA-MRSA clones

**Figure 3.**
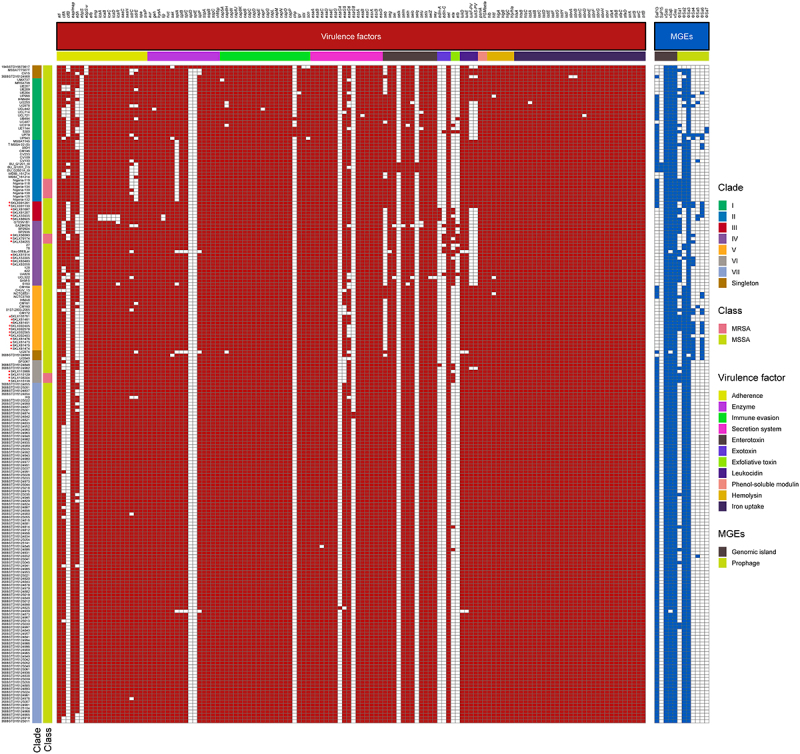
Heatmap of virulence factors and MGEs among the ST121 isolates. The isolates from our collection are indicated by red asterisks.

We next investigated the distribution of virulence factors among global ST121 clones. As shown in (Figure 3), we found that among these clades, there was no significant difference in the virulence genes of ST121 isolates. Specifically, most isolates of each clade (≥90% isolates per clade) harboured virulence genes involved in categories including adhesion, immune evasion, the type VII secretion system, exoenzyme, haemolysin, phenol-soluble modulins, exotoxin (*set*), leukocidin (*lukD* and *lukE*), enterotoxin (*seg*, *sei*, *selm*, *seln*, *selo*, and *selu*) and iron uptake. However, the exfoliative toxin genes *eta* and *etb* were detected in only 29 (14.29%, 29/203) and 9 (4.43%, 9/203) ST121 isolates, respectively, of which 12 and 3 were from China. In addition, 5 Chinese ST121-V isolates carried *eta*, 3 carried *etb*, and 2 carried both toxin genes and belonged to ST121-V-t8660. The EDIN exotoxin gene *edin-A* was only identified in ST121 MSSA isolates from China (except one strain, 3688STDY6124848, from Thailand), and *edin-C* was harboured by the same isolates encoding *etb* (Figure 2 and 3). Of note, most ST121 isolates (164/203, 80.8%) contained PVL. However, the presence of these virulence genes does not necessarily imply their expression, and gene expression levels should be considered when interpreting our results. Further, as shown in **Figure S3A**, the common virulence factors carried by MRSA ST121 and the other epidemic CA-MRSA clones USA300, ST59, and MW2 were similar, except for the enterotoxin gene cluster (egc, including *seg*, *sei*, *selm*, *seln*, *selo* and *selu*), *eta*, *etb*, *edin-C* and *ebpS-v* (a truncated *ebpS* variant with an internal 180 bp deletion) presenting only in ST121 MRSA isolates.

**Figure 4.**
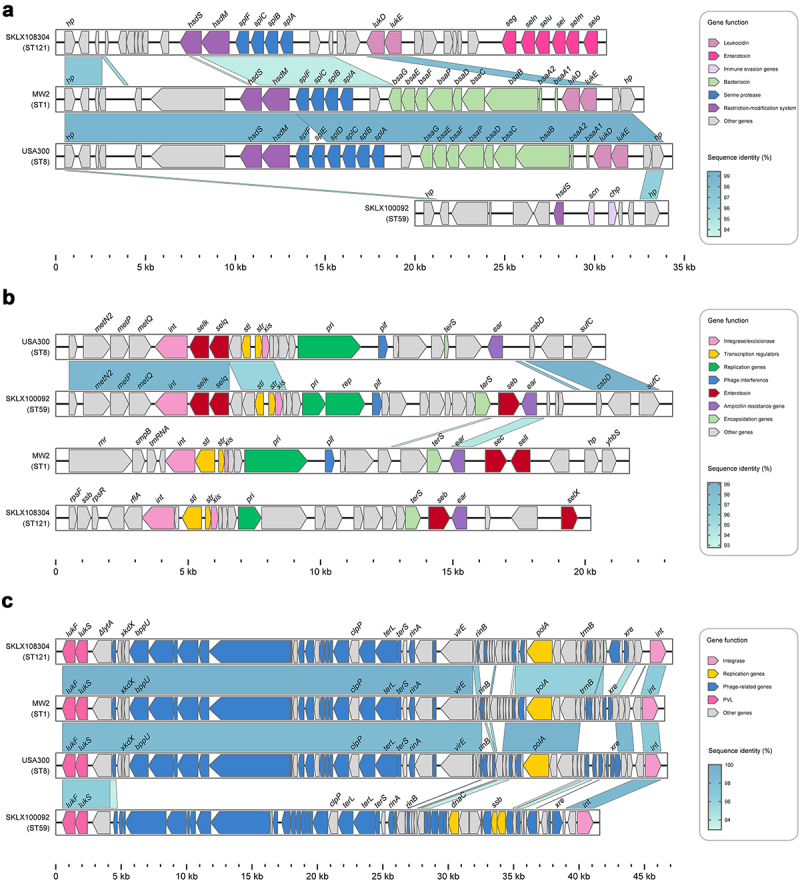
Comparison of pathogenicity islands and prophages among ST121 isolates, and USA300, MW2, and ST59 strains. Comparison of the genomic structure of (a) vSaβ, (b) SaPI and (c) ΦSa2 between ST121 isolates, USA300, MW2, and ST59 strains. Arrowed boxes represent genes that are coloured according to functional classification.

All ST121 isolates carried the vSaα and vSaγ genomic islands as well as the ΦSa3 prophage (except for 3 isolates); and in addition to 4 ST121 isolates within clade I harbouring immune evasion cluster (IEC) type B (encoding *scn-chp-sak*), all other ΦSa3-carrying ST121 isolates contained IEC E (*scn-sak*) (Figure 3). Most vSaβ types in *S. aureus* encode a restriction/modification system (*hsdM*/*hsdS*), a set of *spl* genes, a cluster of *bsa* genes, the *lukD* and *lukE* and egc. Nearly all ST121 isolates (98.52%, 200/203) examined in this study harboured vSaβ, although they all lacked the *bsa* gene cluster compared with CA-MRSA clones MW2 and USA300; however, the egc in ST121 isolates was lacking in the other CA-MRSA clones (Figures 4a). Horizontal genetic transfer by *S. aureus* pathogenicity islands (SaPIs) has played a significant role in the evolution and virulence of *S. aureus* [21]. Most of the ST121 isolates harboured SaPI4 (74.38%, 151/203), which contained *seb* but lacked the *selk* and *selq* genes compared with the SaPI of USA300 and ST59 clones (Figure 4b). The ΦSa2 prophage harbouring bi-component toxin PVL, which is associated with enhanced virulence and epidemic dissemination of CA-MRSA strains [22], was found in 80.8% (164/203) of the ST121 isolates. In addition, ΦSa2 in ST121 shared a larger number of homologous genes with the USA300 and MW2 clones than with ST59 (Figure 4c).

### CA-MRSA ST121 exhibited higher virulence than CA-MRSA ST59 and MW2

**Figure 5.**
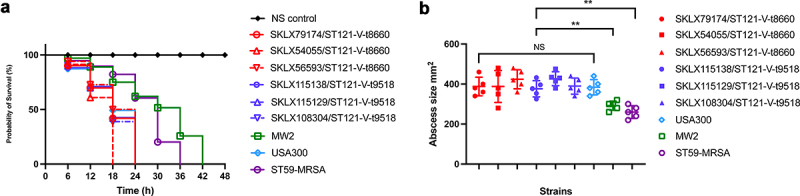
Virulence phenotype of MRSA ST121 strains from our collection, the CA-MRSA clone USA300, USA400 MW2, and ST59. (a) Survival curves of *G. mellonella* larvae (*n* = 20 per strain) infected with strains or sterile PBS. (b) Skin lesion in the mouse skin infection model. Five mice were infected per strain. **, P < 0.01; NS, not significant (*P* ≥ 0.05).

To expand our knowledge of the virulence of other globally occurring CA-MRSA lineages other than the USA300 lineage that has been the focus of most previous research, we here compared the virulence of the ST121-MRSA-V clone with the major CA-MRSA lineages (USA300, MW2 and ST59). As shown in (Figure 5a), in the *G. mellonella* larva infection model, the percentage of surviving larvae infected with ST121 was slightly higher than the percentage of surviving larvae infected with USA300 (*P* > 0.05), indicating that the pathogenicity of ST121-MRSA is similar to that of the CA-MRSA clone USA300, which is a particularly notorious widespread pathogen causing CA-MRSA infections in the USA. However, the percentage of surviving larvae infected with ST121 and USA300 (*G. mellonella* survival = 0% at 24 h) was significantly lower than that of larvae infected with MW2 and ST59 (*G. mellonella* survival ≥ 50% at 24 h), suggesting that the ST121-MRSA clone is more virulent than other CA-MRSA clones, such as ST59 and MW2.

In the mouse skin infection model, skin lesions induced by ST121 were similar in size to those caused by USA300 (*P* > 0.05). However, both ST121 and USA300 resulted in significantly larger lesions compared to those caused by MRSA ST59 and MW2 (*P* < 0.01), indicating that MRSA ST121 possesses high virulence level and can cause more extensive skin damages than other clones in mice (Figure 5b). Based on the abovementioned analyses of virulence factors and MGEs, we speculate that the exclusive virulence genes *eta*, *etb*, *edin-C* and egc (presenting only in ST121), and arginine catabolic mobile element (ACME) (presenting only in USA300) (Figure S3B) might be responsible for the increasing pathogenicity of MRSA.

### CA-MRSA ST121 clone is less efficient in colonization and tolerance than epidemic CA-MRSA clones

**Figure 6.**
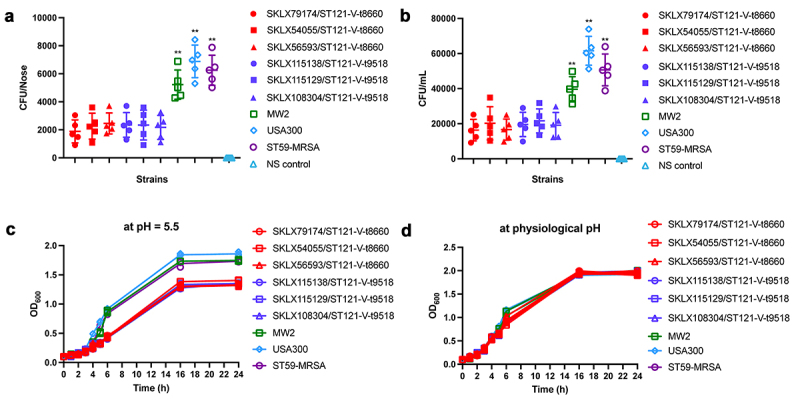
Nasal colonization, cell adhesion capacity, and growth rates of ST121-MRSA isolates from our collection and the CA-MRSA clones USA300, MW2, and ST59. (a) Nasal colonization capacity of strains. Data for each ST121 MRSA isolate in five mice were averaged. (b) Adhesive capacity of tested strains in A549 human alveolar epithelial cells. (c, d) Growth curves of strains in the acid environment (pH 5.5) and physiological environment (pH 7.4). **, P < 0.01.

In this study, we found that ST121-MRSA clone was more virulent than MW2 and ST59 CA-MRSA clones. However, as a globally disseminated clone, ST121 did not achieve the level of epidemiological success of CA-MRSA. These results indicate that the driving factors of dominant CA-MRSA clones include not only their high level of virulence but also other factors such as the colonization ability and physicochemical pressure resistance. Thus, we evaluated the colonization ability of these CA-MRSA clones. There was no difference between CA-MRSA clones in the ability to form a biofilm, as all strains were negative for biofilm formation (data not shown). However, as shown in (Figure 6a), in the nasal colonization model, we found that ST121 exhibited much lower nasal colonization efficiency than the USA300, ST59 and MW2 (*P* < 0.01). Correspondingly, ST121-MRSA also exhibited a lower capacity for epithelial cell adhesion than USA300, ST59 and MW2 (*P* < 0.01, Figure 6b). Interestingly, we found that compared with other CA-MRSA clones, 98.52% (200/203) ST121 including all MRSA strains in this study harboured the truncated derivatives of *ebpS* gene, lacking 180 bp DNA segment flanking the hydrophobic domain H1 (Figure 3 and Figure S4). As shown in (Figure 6c), we found that during the exponential growth phase, ST121 exhibited a reduced growth rate compared with USA300, ST59, and MW2, indicating that ST121 is less tolerant of acid environments than other CA-MRSA clones.

## Discussion

The rising rate of community-associated infection caused by *S. aureus* has aroused global public concern for decades [23]. In recent years, various population genomic studies of CA-SA clonotypes ST8, ST59, ST398, and other community-associated clones have been well performed [24–27], but the genetic characterization of the globally disseminated hypervirulent *S. aureus* clone ST121 is lacking. Therefore, herein, we characterized 28 CA-SA ST121 isolates obtained from BSIs, along with 175 ST121 isolates from the public database.

Our phylogenetic analysis showed that the global ST121 strains from different countries clustered in different clades, suggesting cross-country or even cross-continental transmission. Molecularly, numerous *S. aureus* ST121 isolates contained similar virulence factors associated with MGEs, such as *scn* and *sak* encoded by IEC E within ΦSa3 and PVL encoded by the ΦSa2. By contrast, the *spa* types exhibited extensive diversity, with t159 (*n* = 78), t134 (*n* = 19), t645 (*n* = 16), and t7002 (*n* = 16) being the predominant *spa* types. These general characteristics highlight the consistency and the complexity of global ST121 lineages.

In this study, the virulence of the ST121-MRSA clone is as pronounced as that of the highly virulent USA300 CA-MRSA clone and exceeds that of the predominant geographically matched Chinese CA-MRSA lineage ST59 and the other American CA-MRSA clone MW2. Notably, based on the analysis of virulence genes, *eta*, *etb*, *edin-C*, and egc were only found in ST121, while ACME was only present in USA300. Similar to most toxins of *S. aureus*, the ETs are encoded on MGEs [28]. The *eta* gene, which encodes ETA, is harboured on a 43 kb temperate bacteriophage, ϕETA [29], within the bacterial chromosome. ETB, encoded by *etb*, is located on approximately 38.2–38.5 kb plasmids [30]. These toxins, which belong to a family of trypsin-like serine proteases, can facilitate bacterial spread within the human body due to their capacity to disrupt cell-to-cell adhesion [31]. EDIN-C, encoded by *edin-C*, is a member of the *Clostridium botulinum* C3 exoenzyme family that facilitate bacterial dissemination in tissues, resulting in the formation of infection foci [32,33]. Although the precise role of the egc operon located on the vSaβ genomic island remains unclear, Roetzer et al. have proved the toxicity of individual egc members in a rabbit model [34]. Thus, the high virulence of ST121 may be attributed to the combination of these virulence factors encoded by MGEs, such as PVL, *eta*, *etb*, *edin-C*, and egc.

For many pathogens, virulence is positively associated with transmissibility. For instance, the risk of HIV transmission is increased when viral load in the infected host is at its highest level [35]. However, as a globally disseminated hypervirulent *S. aureus* clone, ST121 has not achieved the epidemiological success of other *S. aureus* clones. Experiments in mice nasal and human alveolar epithelial cells showed that ST121 has a much lower host colonization capacity than that of USA300, ST59, and MW2. Comparative genomic analysis between ST121 and other CA-MRSA clones showed that most ST121 isolates (98.52%, 200/203) contained truncated *ebpS* genes, including deletion of the entire hydrophobic domain H1 (Figure 3 and Figure S4). The elastin-binding protein of *Staphylococcus aureus* (EbpS), encoded by *ebpS*, is a 25-kDa protein that may promote the colonization of connective tissues abundant in elastin, such as skin and lung tissue [36]. Therefore, the disappearance of H1 within EbpS might affect the attachment to host cells of ST121-MRSA isolates. Notably, the three ST121-MSSA isolates carrying the *ebpS* gene were located within the basal clade of the ST121-core-genome based phylogenetic tree, indicating subsequent loss of the sequences within *ebpS* in the evolutionary history of ST121 clones (Figure 3).

Due to the low sweat pH and lack of nutrients, the surface of human skin is a harsh acidic environment [37]. Thus, the ability to combat the high levels organic acids in skin could promote bacterial colonization and survival. In this study, under acidic conditions, ST121 exhibited much lower tolerance than USA300, ST59, and MW2. Due to its low colonization ability and low acidic tolerance, ST121 may not outcompete other CA-MRSA clones to become the dominate lineage.

This study has several limitations. First, the utilization of draft genomes for ST121 clones could potentially influence our analysis due to the presence of gaps and errors. Second, the number of ST121 isolates collected was not large enough, which may affect our comprehensive understanding of this lineage. Future studies with larger sample sizes and more comprehensive genomic data would be beneficial for a deeper understanding of the ST121 lineage and its pathogenicity.

## Conclusion

This is the first detailed genomic characterization of global ST121 clones and Chinese ST121 strains isolated from severe infection cases. Our data suggest that ST121-MRSA clones from China may be divided into two distinct lineages with different divergence time and the Asian ST121 isolates have a most likely origin in China. The virulence genes and MGEs were found to be highly similar among isolates from each phylogenetic clade. Furthermore, the results of our investigation support the idea that the capacity of ST121 isolates to cause community-associated infections is possibly due to their high virulence. Nevertheless, being too virulent is still detrimental, as the ST121 clone kills or immobilizes the host before transmission to another host. Our results suggest the inability of ST121 clones to become the dominate lineage correlates to their lower host colonization capacity and acid tolerance compared to other CA-MRSA clones, elucidating the critical role of colonization and environmental adaption in MRSA epidemiological success.

## Supplementary Material

Supplemental Material
