# Supplementary material for: Comparative analysis of genomic characteristics, virulence and fitness of community-associated Staphylococcus aureus ST121 clone causing fatal diseases in China and other CA-MRSA clones
Source: Virulence. 2023 Aug 3;14(1):2242547. doi: 10.1080/21505594.2023.2242547 (PMC10402838; doi:10.1080/21505594.2023.2242547)
Supplement: Supplemental Material [file KVIR_A_2242547_SM5961.zip › Supplemental Material (4).docx]

| **Table S1. Information of 203 ST121 isolates analyzed in this study.** | | | | | | | | |
| --- | --- | --- | --- | --- | --- | --- | --- | --- |
| Strain | GenBank/SRA accession | Length | Year of isolation | Country | Host | Isolation source | *spa* type | SCC*mec* type |
| BU_G1201_t8 | GCA_001297445.1 | 2759850 | 2013 | Ghana | Human | Wound | t314 | MSSA |
| BU_G2601A_t9 | GCA_001297475.1 | 2750732 | 2013 | Ghana | Human | Wound | t314 | MSSA |
| BU_G1201_t13 | GCA_001297535.1 | 2759916 | 2013 | Ghana | Human | Wound | t314 | MSSA |
| XQ | GCA_001444345.1 | 2803594 | 2009 | China | Human | Blood | t159 | MSSA |
| W08A_161214 | GCA_002905405.1 | 2728761 | 2014 | Ghana | Human | Wound | t314 | MSSA |
| W08B_161214 | GCA_002905575.1 | 2728556 | 2014 | Ghana | Human | Wound | t314 | MSSA |
| G703N1B1 | GCA_002934135.2 | 2806379 | 2017 | South Africa | Human | Respiratory tract | t272 | MSSA |
| SAMNA5 | GCA_003038615.1 | 2796695 | 2016 | Lebanon | Human | Skin/soft tissue | t159 | MSSA |
| CM172 | GCA_003236825.1 | 2762507 | 2014 | Italy | Human | Skin/soft tissue | t2530 | MSSA |
| CM164 | GCA_003236935.1 | 2791197 | 2013 | Italy | Human | Skin/soft tissue | t159 | MSSA |
| CM161 | GCA_003236985.1 | 2817926 | 2013 | Italy | Human | Skin/soft tissue | t1211 | MSSA |
| CM160 | GCA_003237005.1 | 2808882 | 2013 | Italy | Human | Skin/soft tissue | t284 | MSSA |
| CM146 | GCA_003237145.1 | 2755498 | 2014 | Italy | Human | Skin/soft tissue | t314 | MSSA |
| 27 | GCA_003354545.1 | 2814328 | 1994 | Germany | Human | Blood | t916 | MSSA |
| 422 | GCA_003354605.1 | 2824814 | 1994 | Germany | Human | Respiratory tract | t162 | MSSA |
| 128 | GCA_003354645.1 | 2824816 | 1994 | Germany | Human | Blood | t162 | MSSA |
| 78 | GCA_003354905.1 | 2853623 | 1994 | Germany | Human | Respiratory tract | t916 | MSSA |
| SauNA3893Lar | GCA_003605275.1 | 2845940 | 2014 | Greece | Human | NA | t162 | MSSA |
| 0137NA2003NA2005 | GCA_005774675.1 | 2824683 | 2005 | Russia | Human | Blood | t435 | MSSA |
| MRSA738 | GCA_009456545.1 | 2798198 | 2016 | USA | Human | Wound | t6465 | MSSA |
| UA828 | GCA_009676125.1 | 2814267 | 2013 | Latin America | Human | Blood | t645 | MSSA |
| SA29KEN | GCA_009690605.1 | 2834535 | 2015 | Kenya | Human | NA | t272 | MSSA |
| 5300 | GCA_010571125.1 | 2846135 | 2018 | Latin America | Human | Blood | t645 | MSSA |
| UCL322 | GCA_011022485.1 | 2757608 | 2012 | Latin America | Human | Blood | t159 | MSSA |
| UC487 | GCA_011022495.1 | 2752075 | 2012 | Latin America | Human | Blood | t2155 | MSSA |
| UCL442 | GCA_011023125.1 | 2757955 | 2012 | Latin America | Human | Blood | t645 | MSSA |
| UE207 | GCA_011023495.1 | 2786964 | 2012 | Latin America | Human | Blood | NA | MSSA |
| UCL721 | GCA_011024035.1 | 2787892 | 2014 | Latin America | Human | Blood | t645 | MSSA |
| UG974 | GCA_011024285.1 | 2757465 | 2012 | Latin America | Human | Blood | t1425 | MSSA |
| UG978 | GCA_011024405.1 | 2835367 | 2013 | Latin America | Human | Blood | t645 | MSSA |
| UP943 | GCA_011024635.1 | 2733421 | 2013 | Latin America | Human | Blood | t645 | MSSA |
| UP998 | GCA_011024705.1 | 2805247 | 2013 | Latin America | Human | Blood | t645 | MSSA |
| UG949 | GCA_011025585.1 | 2783007 | 2012 | Latin America | Human | Blood | t159 | MSSA |
| UMX737 | GCA_011025765.1 | 2790749 | 2012 | Latin America | Human | Blood | t6465 | MSSA |
| UE1144 | GCA_011026015.1 | 2789568 | 2013 | Latin America | Human | Blood | t645 | MSSA |
| UE209 | GCA_011026045.1 | 2825496 | 2012 | Latin America | Human | Blood | t6465 | MSSA |
| UC319 | GCA_011026195.1 | 2779861 | 2012 | Latin America | Human | Blood | t2155 | MSSA |
| UG250 | GCA_011026885.1 | 2819941 | 2012 | Latin America | Human | Blood | t645 | MSSA |
| UE204 | GCA_011028335.1 | 2776515 | 2012 | Latin America | Human | Blood | t6465 | MSSA |
| UB494 | GCA_011030455.1 | 2828401 | 2012 | Latin America | Human | Blood | t2651 | MSSA |
| UP79 | GCA_011031025.1 | 2819151 | 2011 | Latin America | Human | Blood | t645 | MSSA |
| UCL714 | GCA_011031855.1 | 2726686 | 2014 | Latin America | Human | Blood | t645 | MSSA |
| CV109 | GCA_012652355.1 | 2787797 | 1997 | Cape Verde | Human | Respiratory tract | t314 | MSSA |
| CV133 | GCA_012652495.1 | 2835823 | 1997 | Cape Verde | Human | Respiratory tract | t314 | MSSA |
| CV15 | GCA_012652545.1 | 2786059 | 1997 | Cape Verde | Human | Respiratory tract | t940 | MSSA |
| CV233 | GCA_012652885.1 | 2762039 | 2013 | Cape Verde | Human | Respiratory tract | t314 | MSSA |
| NigeriaNA134 | GCA_013345465.1 | 2812146 | 2016 | Nigeria | Food | Food | t314 | IVa (2B) |
| NigeriaNA138 | GCA_013345485.1 | 2807757 | 2016 | Nigeria | Food | Food | t314 | IVa (2B) |
| NigeriaNA139 | GCA_013345495.1 | 2813024 | 2016 | Nigeria | Food | Food | t314 | IVa (2B) |
| NigeriaNA118 | GCA_013345575.1 | 2808100 | 2016 | Nigeria | Food | Food | t314 | IVa (2B) |
| NigeriaNA128 | GCA_013345585.1 | 2812772 | 2016 | Nigeria | Food | Food | t314 | IVa (2B) |
| NigeriaNA132 | GCA_013345595.1 | 2786904 | 2016 | Nigeria | Food | Food | t314 | MSSA |
| NigeriaNA119 | GCA_013345645.1 | 2812805 | 2016 | Nigeria | Food | Food | t314 | IVa (2B) |
| 6160 | GCA_017582155.1 | 2772609 | 2016 | France | Human | Blood | t159 | MSSA |
| IHMA84 | GCA_017695745.1 | 2801800 | 2015 | USA | Human | Eye | t645 | MSSA |
| IHMA8 | GCA_017695845.1 | 2816186 | 2009 | Israel | Human | Eye | t3454 | MSSA |
| SP2936 | GCA_017838335.1 | 2781547 | 2020 | India | Human | Respiratory tract | t272 | MSSA |
| SP3067 | GCA_017838375.1 | 2785949 | 2020 | India | Human | Respiratory tract | t17234 | MSSA |
| SP2924 | GCA_017838485.1 | 2780717 | 2020 | India | Human | Respiratory tract | t272 | MSSA |
| 1943STDY5573617 | GCA_900070305.1 | 2678107 | 1999 | United Kingdom | Animal | Animal | t645 | MSSA |
| 080H | GCA_900097485.1 | 2768538 | 2014 | Tanzania | Human | Respiratory tract | t314 | MSSA |
| MSSA777507T | GCA_900097855.1 | 2823466 | 2013 | Tanzania | Human | Wound | t645 | MSSA |
| MSSAT145 | GCA_900097865.1 | 2769569 | 2005 | Tanzania | Human | Respiratory tract | t314 | MSSA |
| T MSSA 02 (S) | GCA_900097915.1 | 2815452 | 2013 | Tanzania | Human | Wound | t314 | MSSA |
| 3688STDY6124973 | GCA_900124675.1 | 2796552 | 2015 | Thailand | Human | Unknown sterile site | t159 | MSSA |
| 3688STDY6124975 | GCA_900124695.1 | 2807081 | 2015 | Thailand | Human | Unknown sterile site | t159 | MSSA |
| 3688STDY6124976 | GCA_900124705.1 | 2797178 | 2015 | Thailand | Human | Unknown sterile site | t159 | MSSA |
| 3688STDY6124977 | GCA_900124715.1 | 2799775 | 2015 | Thailand | Human | Unknown sterile site | t159 | MSSA |
| 3688STDY6124961 | GCA_900124725.1 | 2796148 | 2015 | Thailand | Human | Unknown sterile site | t159 | MSSA |
| 3688STDY6124979 | GCA_900124735.1 | 2798059 | 2015 | Thailand | Human | Unknown sterile site | t7002 | MSSA |
| 3688STDY6124980 | GCA_900124745.1 | 2799853 | 2015 | Thailand | Human | Unknown sterile site | t159 | MSSA |
| 3688STDY6124982 | GCA_900124765.1 | 2837660 | 2015 | Thailand | Human | Unknown sterile site | t159 | MSSA |
| 3688STDY6124962 | GCA_900124815.1 | 2832226 | 2015 | Thailand | Human | Unknown sterile site | t3204 | MSSA |
| 3688STDY6124989 | GCA_900124825.1 | 2797965 | 2015 | Thailand | Human | Unknown sterile site | t159 | MSSA |
| 3688STDY6124992 | GCA_900124845.1 | 2800362 | 2015 | Thailand | Human | Unknown sterile site | t159 | MSSA |
| 3688STDY6124996 | GCA_900124865.1 | 2794353 | 2015 | Thailand | Human | Unknown sterile site | t159 | MSSA |
| 3688STDY6124997 | GCA_900124875.1 | 2835790 | 2015 | Thailand | Human | Unknown sterile site | t162 | MSSA |
| 3688STDY6124963 | GCA_900124895.1 | 2793939 | 2015 | Thailand | Human | Unknown sterile site | t159 | MSSA |
| 3688STDY6124999 | GCA_900124905.1 | 2784456 | 2015 | Thailand | Human | Unknown sterile site | t159 | MSSA |
| 3688STDY6125001 | GCA_900124925.1 | 2794283 | 2015 | Thailand | Human | Unknown sterile site | t159 | MSSA |
| 3688STDY6125007 | GCA_900124955.1 | 2793830 | 2015 | Thailand | Human | Unknown sterile site | t159 | MSSA |
| 3688STDY6125011 | GCA_900125005.1 | 2829727 | 2015 | Thailand | Human | Unknown sterile site | t159 | MSSA |
| 3688STDY6125012 | GCA_900125015.1 | 2795999 | 2015 | Thailand | Human | Unknown sterile site | t284 | MSSA |
| 3688STDY6125013 | GCA_900125025.1 | 2796788 | 2015 | Thailand | Human | Unknown sterile site | t159 | MSSA |
| 3688STDY6125018 | GCA_900125065.1 | 2810664 | 2015 | Thailand | Human | Unknown sterile site | t159 | MSSA |
| 3688STDY6125019 | GCA_900125085.1 | 2801390 | 2015 | Thailand | Human | Unknown sterile site | t7002 | MSSA |
| 3688STDY6124965 | GCA_900125095.1 | 2793876 | 2015 | Thailand | Human | Unknown sterile site | t159 | MSSA |
| 3688STDY6125022 | GCA_900125105.1 | 2795734 | 2015 | Thailand | Human | Unknown sterile site | t159 | MSSA |
| 3688STDY6125024 | GCA_900125125.1 | 2789258 | 2015 | Thailand | Human | Unknown sterile site | t159 | MSSA |
| 3688STDY6124966 | GCA_900125175.1 | 2792148 | 2015 | Thailand | Human | Unknown sterile site | t159 | MSSA |
| 3688STDY6125033 | GCA_900125205.1 | 2800319 | 2015 | Thailand | Human | Unknown sterile site | t159 | MSSA |
| 3688STDY6125035 | GCA_900125225.1 | 2799344 | 2015 | Thailand | Human | Unknown sterile site | t159 | MSSA |
| 3688STDY6125036 | GCA_900125235.1 | 2838102 | 2015 | Thailand | Human | Unknown sterile site | t159 | MSSA |
| 3688STDY6125037 | GCA_900125245.1 | 2799250 | 2015 | Thailand | Human | Unknown sterile site | t159 | MSSA |
| 3688STDY6125039 | GCA_900125265.1 | 2794374 | 2015 | Thailand | Human | Unknown sterile site | t159 | MSSA |
| 3688STDY6124967 | GCA_900125275.1 | 2792074 | 2015 | Thailand | Human | Unknown sterile site | t159 | MSSA |
| 3688STDY6125041 | GCA_900125285.1 | 2799823 | 2015 | Thailand | Human | Unknown sterile site | t7002 | MSSA |
| 3688STDY6125042 | GCA_900125295.1 | 2794024 | 2015 | Thailand | Human | Unknown sterile site | t159 | MSSA |
| 3688STDY6125045 | GCA_900125315.1 | 2844797 | 2015 | Thailand | Human | Unknown sterile site | t7002 | MSSA |
| 3688STDY6124968 | GCA_900125355.1 | 2818134 | 2015 | Thailand | Human | Unknown sterile site | t9090 | MSSA |
| 3688STDY6124812 | GCA_900125365.1 | 2801662 | 2015 | Thailand | Human | Unknown sterile site | t272 | MSSA |
| 3688STDY6124814 | GCA_900125375.1 | 2842272 | 2015 | Thailand | Human | Unknown sterile site | t159 | MSSA |
| 3688STDY6124818 | GCA_900125405.1 | 2795145 | 2015 | Thailand | Human | Unknown sterile site | t159 | MSSA |
| 3688STDY6124820 | GCA_900125425.1 | 2800554 | 2015 | Thailand | Human | Unknown sterile site | t7002 | MSSA |
| 3688STDY6124821 | GCA_900125435.1 | 2793891 | 2015 | Thailand | Human | Unknown sterile site | t159 | MSSA |
| 3688STDY6124824 | GCA_900125455.1 | 2797355 | 2015 | Thailand | Human | Unknown sterile site | t159 | MSSA |
| 3688STDY6124825 | GCA_900125465.1 | 2793050 | 2015 | Thailand | Human | Unknown sterile site | t159 | MSSA |
| 3688STDY6124827 | GCA_900125485.1 | 2789391 | 2015 | Thailand | Human | Unknown sterile site | t159 | MSSA |
| 3688STDY6124829 | GCA_900125495.1 | 2795596 | 2015 | Thailand | Human | Unknown sterile site | t159 | MSSA |
| 3688STDY6124834 | GCA_900125515.1 | 2804431 | 2015 | Thailand | Human | Unknown sterile site | t272 | MSSA |
| 3688STDY6124833 | GCA_900125535.1 | 2787623 | 2015 | Thailand | Human | Unknown sterile site | t1425 | MSSA |
| 3688STDY6124837 | GCA_900125565.1 | 2795347 | 2015 | Thailand | Human | Unknown sterile site | t159 | MSSA |
| 3688STDY6124838 | GCA_900125575.1 | 2797263 | 2015 | Thailand | Human | Unknown sterile site | t159 | MSSA |
| 3688STDY6124840 | GCA_900125585.1 | 2793558 | 2015 | Thailand | Human | Unknown sterile site | t4499 | MSSA |
| 3688STDY6124841 | GCA_900125595.1 | 2827945 | 2015 | Thailand | Human | Unknown sterile site | t159 | MSSA |
| 3688STDY6124843 | GCA_900125605.1 | 2812935 | 2015 | Thailand | Human | Unknown sterile site | t8081 | MSSA |
| 3688STDY6124848 | GCA_900125645.1 | 2852478 | 2015 | Thailand | Human | Unknown sterile site | t3204 | MSSA |
| 3688STDY6124849 | GCA_900125655.1 | 2801060 | 2015 | Thailand | Human | Unknown sterile site | t159 | MSSA |
| 3688STDY6124852 | GCA_900125675.1 | 2844417 | 2015 | Thailand | Human | Unknown sterile site | t7002 | MSSA |
| 3688STDY6124854 | GCA_900125685.1 | 2796177 | 2015 | Thailand | Human | Unknown sterile site | t159 | MSSA |
| 3688STDY6124855 | GCA_900125695.1 | 2782953 | 2015 | Thailand | Human | Unknown sterile site | t159 | MSSA |
| 3688STDY6124885 | GCA_900125855.1 | 2797173 | 2015 | Thailand | Human | Unknown sterile site | t159 | MSSA |
| 3688STDY6124886 | GCA_900125865.1 | 2842678 | 2015 | Thailand | Human | Unknown sterile site | t7002 | MSSA |
| 3688STDY6124890 | GCA_900125895.1 | 2734927 | 2015 | Thailand | Human | Unknown sterile site | t645 | MSSA |
| 3688STDY6124873 | GCA_900125905.1 | 2799366 | 2015 | Thailand | Human | Unknown sterile site | t159 | MSSA |
| 3688STDY6124898 | GCA_900125935.1 | 2792144 | 2015 | Thailand | Human | Unknown sterile site | t645 | MSSA |
| 3688STDY6124900 | GCA_900125955.1 | 2808879 | 2015 | Thailand | Human | Unknown sterile site | t159 | MSSA |
| 3688STDY6124874 | GCA_900125965.1 | 2794649 | 2015 | Thailand | Human | Unknown sterile site | t159 | MSSA |
| 3688STDY6124901 | GCA_900125975.1 | 2799483 | 2015 | Thailand | Human | Unknown sterile site | t9090 | MSSA |
| 3688STDY6124905 | GCA_900126015.1 | 2795262 | 2015 | Thailand | Human | Unknown sterile site | t272 | MSSA |
| 3688STDY6124908 | GCA_900126035.1 | 2705196 | 2015 | Thailand | Human | Unknown sterile site | t284 | MSSA |
| 3688STDY6124919 | GCA_900126125.1 | 2792375 | 2015 | Thailand | Human | Unknown sterile site | t159 | MSSA |
| 3688STDY6124931 | GCA_900126235.1 | 2795897 | 2015 | Thailand | Human | Unknown sterile site | t7002 | MSSA |
| 3688STDY6124935 | GCA_900126285.1 | 2799945 | 2015 | Thailand | Human | Unknown sterile site | t159 | MSSA |
| 3688STDY6124936 | GCA_900126295.1 | 2796554 | 2015 | Thailand | Human | Unknown sterile site | t159 | MSSA |
| 3688STDY6124939 | GCA_900126325.1 | 2779584 | 2015 | Thailand | Human | Unknown sterile site | t159 | MSSA |
| 3688STDY6124878 | GCA_900126335.1 | 2798651 | 2015 | Thailand | Human | Unknown sterile site | t7002 | MSSA |
| 3688STDY6124943 | GCA_900126355.1 | 2798819 | 2015 | Thailand | Human | Unknown sterile site | t159 | MSSA |
| 3688STDY6124946 | GCA_900126385.1 | 2796159 | 2015 | Thailand | Human | Unknown sterile site | t159 | MSSA |
| 3688STDY6124949 | GCA_900126405.1 | 2793693 | 2015 | Thailand | Human | Unknown sterile site | t159 | MSSA |
| 3688STDY6124952 | GCA_900126425.1 | 2790142 | 2015 | Thailand | Human | Unknown sterile site | t159 | MSSA |
| 3688STDY6124953 | GCA_900126435.1 | 2801713 | 2015 | Thailand | Human | Unknown sterile site | t7002 | MSSA |
| 3688STDY6124956 | GCA_900126465.1 | 2800896 | 2015 | Thailand | Human | Unknown sterile site | t272 | MSSA |
| 3688STDY6124957 | GCA_900126475.1 | 2797538 | 2015 | Thailand | Human | Unknown sterile site | t159 | MSSA |
| 3688STDY6125051 | GCA_900126635.1 | 2796255 | 2015 | Thailand | Human | Unknown sterile site | t159 | MSSA |
| 3688STDY6125052 | GCA_900126715.1 | 2795913 | 2015 | Thailand | Human | Unknown sterile site | t159 | MSSA |
| 3688STDY6125104 | GCA_900126765.1 | 2801380 | 2015 | Thailand | Human | Unknown sterile site | t9090 | MSSA |
| 3688STDY6125054 | GCA_900126845.1 | 2800266 | 2015 | Thailand | Human | Unknown sterile site | t7002 | MSSA |
| 3688STDY6125055 | GCA_900126925.1 | 2790813 | 2015 | Thailand | Human | Unknown sterile site | t159 | MSSA |
| 3688STDY6125141 | GCA_900127095.1 | 2805494 | 2015 | Thailand | Human | Unknown sterile site | t7002 | MSSA |
| 3688STDY6124960 | GCA_900127635.1 | 2792111 | 2015 | Thailand | Human | Unknown sterile site | t159 | MSSA |
| 3688STDY6124985 | GCA_900127685.1 | 2802783 | 2015 | Thailand | Human | Unknown sterile site | t7002 | MSSA |
| 3688STDY6124991 | GCA_900127705.1 | 2799915 | 2015 | Thailand | Human | Unknown sterile site | t159 | MSSA |
| 3688STDY6124994 | GCA_900127715.1 | 2788066 | 2015 | Thailand | Human | Unknown sterile site | t159 | MSSA |
| 3688STDY6125021 | GCA_900127745.1 | 2790981 | 2015 | Thailand | Human | Unknown sterile site | t7002 | MSSA |
| 3688STDY6125028 | GCA_900127755.1 | 2792830 | 2015 | Thailand | Human | Unknown sterile site | t159 | MSSA |
| 3688STDY6125030 | GCA_900127765.1 | 2837051 | 2015 | Thailand | Human | Unknown sterile site | t159 | MSSA |
| 3688STDY6125040 | GCA_900127775.1 | 2796813 | 2015 | Thailand | Human | Unknown sterile site | t159 | MSSA |
| 3688STDY6125044 | GCA_900127785.1 | 2796341 | 2015 | Thailand | Human | Unknown sterile site | t159 | MSSA |
| 3688STDY6124842 | GCA_900127855.1 | 2790701 | 2015 | Thailand | Human | Unknown sterile site | t159 | MSSA |
| 3688STDY6124846 | GCA_900127865.1 | 2800129 | 2015 | Thailand | Human | Unknown sterile site | t7002 | MSSA |
| 3688STDY6124856 | GCA_900127885.1 | 2810322 | 2015 | Thailand | Human | Unknown sterile site | NA | MSSA |
| 3688STDY6124867 | GCA_900127905.1 | 2799126 | 2015 | Thailand | Human | Unknown sterile site | t159 | MSSA |
| 3688STDY6124881 | GCA_900127925.1 | 2802028 | 2015 | Thailand | Human | Unknown sterile site | t2086 | MSSA |
| 3688STDY6124882 | GCA_900127945.1 | 2800846 | 2015 | Thailand | Human | Unknown sterile site | t7002 | MSSA |
| 3688STDY6124893 | GCA_900127985.1 | 2796744 | 2015 | Thailand | Human | Unknown sterile site | t159 | MSSA |
| 3688STDY6124909 | GCA_900128015.1 | 2797560 | 2015 | Thailand | Human | Unknown sterile site | t159 | MSSA |
| 3688STDY6124941 | GCA_900128075.1 | 2793982 | 2015 | Thailand | Human | Unknown sterile site | t8081 | MSSA |
| 3688STDY6124948 | GCA_900128085.1 | 2799139 | 2015 | Thailand | Human | Unknown sterile site | t159 | MSSA |
| 3688STDY6124951 | GCA_900128105.1 | 2798967 | 2015 | Thailand | Human | Unknown sterile site | t159 | MSSA |
| 3688STDY6125058 | GCA_900128125.1 | 2792970 | 2015 | Thailand | Human | Unknown sterile site | t159 | MSSA |
| 3688STDY6125061 | GCA_900128135.1 | 2796164 | 2015 | Thailand | Human | Unknown sterile site | t272 | MSSA |
| CHUV_13 | GCA_900155755.1 | 2785685 | 2014 | Switzerland | Human | Skin/soft tissue | t272 | MSSA |
| NCTC8531 | GCA_900457405.1 | 2865203 | 1935 | United Kingdom | Human | Blood | NA | MSSA |
| NCTC3750 | GCA_900457935.1 | 2892877 | 1932 | United Kingdom | Human | Skin/soft tissue | NA | MSSA |
| SKLX55935 | JAJPPL000000000 | 2830811 | 2016 | China | Human | Blood | t2091 | MSSA |
| SKLX61207 | JAJPPK000000000 | 2862041 | 2017 | China | Human | Blood | t2019 | MSSA |
| SKLX61451 | JAJPPJ000000000 | 2819400 | 2017 | China | Human | Blood | t435 | MSSA |
| SKLX61461 | JAJPPI000000000 | 2819766 | 2017 | China | Human | Blood | t435 | MSSA |
| SKLX61473 | JAJPPH000000000 | 2782618 | 2017 | China | Human | Blood | t2391 | MSSA |
| SKLX61474 | JAJPPG000000000 | 2782615 | 2017 | China | Human | Blood | t2391 | MSSA |
| SKLX61475 | JAJPPF000000000 | 2782779 | 2017 | China | Human | Blood | t2391 | MSSA |
| SKLX61476 | JAJPPE000000000 | 2782734 | 2017 | China | Human | Blood | t2391 | MSSA |
| SKLX61697 | JAJPPD000000000 | 2861785 | 2017 | China | Human | Blood | t2019 | MSSA |
| SKLX63483 | JAJJDW000000000 | 2836237 | 2018 | China | Human | Blood | t2087 | MSSA |
| SKLX63558 | JAJJDY000000000 | 2836287 | 2018 | China | Human | Blood | t2087 | MSSA |
| SKLX51514 | JAJJGX000000000 | 2794605 | 2014 | China | Human | Blood | t2092 | MSSA |
| SKLX53305 | JAJJHA000000000 | 2793606 | 2015 | China | Human | Blood | t1425 | MSSA |
| SKLX54055 | SRR15460898 | 2901193 | 2015 | China | Human | Blood | t8660 | V (5C2&5) |
| SKLX56593 | SRR15460427 | 2897504 | 2016 | China | Human | Blood | t8660 | V (5C2&5) |
| SKLX79174 | SRR15460559 | 2815151 | 2018 | China | Human | Blood | t8660 | V (5C2&5) |
| SKLX108304 | SRR15461117 | 2876701 | 2019 | China | Human | Blood | t9518 | V (5C2&5) |
| SKLX115129 | SRR15460533 | 2905627 | 2019 | China | Human | Blood | t9518 | V (5C2&5) |
| SKLX115138 | SRR15460530 | 2877730 | 2019 | China | Human | Blood | t9518 | V (5C2&5) |
| SKLX105761 | JAJPPC000000000 | 2816378 | 2019 | China | Human | Blood | t435 | MSSA |
| SKLX113906 | JAJPPB000000000 | 2836377 | 2019 | China | Human | Blood | t159 | MSSA |
| SKLX88925 | JAJPPA000000000 | 2789214 | 2019 | China | Human | Blood | NA | MSSA |
| SKLX001130 | JAJPOZ000000000 | 2762880 | 2011 | China | Human | Blood | t162 | MSSA |
| SKLX001201 | JAJPOY000000000 | 2763366 | 2011 | China | Human | Blood | t162 | MSSA |
| SKLX002403 | JAJPOX000000000 | 2833164 | 2011 | China | Human | Blood | t435 | MSSA |
| SKLX002405 | JAJPOW000000000 | 2816787 | 2011 | China | Human | Blood | t435 | MSSA |
| SKLX002565 | JAJPOV000000000 | 2767497 | 2011 | China | Human | Blood | t284 | MSSA |
| SKLX002578 | JAJPOU000000000 | 2835596 | 2011 | China | Human | Blood | t7065 | MSSA |

NA, not available.

**Table S2. Brief clinical records of CA-SA infections caused by ST121 isolates.**

| **Strains ID/Case** | **Separation time** | **Region** | **Age** | **Sex** | **Clinical diagnosis** | **Outcome** |
| --- | --- | --- | --- | --- | --- | --- |
| SKLX001130 | 2011 | Guangdong | 33 | Male | Skin abscess | Cured |
| SKLX001201 | 2011 | Guangdong | 28 | Female | Skin abscess | Cured |
| SKLX002403 | 2011 | Xinjiang | 41 | Female | Folliculitis | Cured |
| SKLX002405 | 2011 | Xinjiang | 37 | Male | Cellulitis | Cured |
| SKLX002565 | 2011 | Xinjiang | 61 | Female | Pneumonia | Death |
| SKLX002578 | 2011 | Xinjiang | 17 | Male | Furuncle | Cured |
| SKLX51514 | 2014 | Jiangsu | 6 | Female | SSSS | Cured |
| SKLX53305 | 2015 | Zhejiang | 5 | Female | Skin abscess | Death |
| SKLX54055 | 2015 | Anhui | 35 | Male | Impetigo | Cured |
| SKLX55935 | 2016 | Shandong | 54 | Female | Skin abscess | Death |
| SKLX56593 | 2016 | Fujian | 2 weeks | Male | SSSS | Death |
| SKLX61207 | 2017 | Henan | 43 | Female | Impetigo | Cured |
| SKLX61451 | 2017 | Xinjiang | 59 | Female | Pneumonia | Cured |
| SKLX61461 | 2017 | Xinjiang | 72 | Male | Pneumonia | Cured |
| SKLX61473 | 2017 | Xinjiang | 63 | Female | Pneumonia | Cured |
| SKLX61474 | 2017 | Xinjiang | 55 | Male | Pneumonia | Cured |
| SKLX61475 | 2017 | Xinjiang | 61 | Female | Pneumonia | Cured |
| SKLX61476 | 2017 | Xinjiang | 45 | Female | Pneumonia | Cured |
| SKLX61697 | 2017 | Henan | 35 | Male | Impetigo | Cured |
| SKLX63483 | 2018 | Anhui | 2 | Female | SSSS | Cured |
| SKLX63558 | 2018 | Anhui | 3 | Male | SSSS | Cured |
| SKLX79174 | 2018 | Hunan | 5 | Female | SSSS | Cured |
| SKLX108304 | 2019 | Jiangxi | 3 weeks | Male | SSSS | Cured |
| SKLX115129 | 2019 | Jiangxi | 2 weeks | Female | SSSS | Cured |
| SKLX115138 | 2019 | Jiangxi | 2 weeks | Male | SSSS | Cured |
| SKLX105761 | 2019 | Gansu | 4 months | Female | Pneumonia | Death |
| SKLX113906 | 2019 | Hubei | 66 | Male | Pneumonia | Death |
| SKLX88925 | 2019 | Hunan | 23 | Female | Impetigo | Cured |

**SSSS, staphylococcal scalded skin syndrome.**





**Figure S1.** A dated genome-wide SNP phylogeny of ST121 isolates. The divergence time and 95% HPD intervals of main clades are shown at the respective nodes. The ST121-MRSA isolates from China are indicated by red boxes, with relevant divergence time and 95% HPD intervals shown nearby the black arrows.





**Figure S2.** Phylogeographic analysis of globalST121 isolates based on the Bayesian Binary MCMC method in RASP v4.2.

**

**

**Figure S3.** Network diagram of (A) all virulence genes (VGs) and (B) MGEs identified among ST121-MRSA isolates and CA-MRSA clones USA300, MW2, and ST59. “Shared” indicates VGs or MGEs are present in all MRSA clones, “Accessory” indicates VGs or MGEs present in two MRSA clones, and “ST-specific” indicates VGs or MGEs present in specific MRSA clone.





Figure S4. Amino acid sequence alignment of *ebpS* genes among ST121-MRSA isolates and CA-MRSA clones USA300, MW2, and ST59. Dots stand for amino acid residues identical to those of *ebpS* of USA300. The deleted amino acid residues in the ST121 are shaded in gray. The predicated hydrophobic domains H1-H3 are indicated above the corresponding sequences.
