# Supplementary material for: Comparative analysis of genomic characteristics, virulence and fitness of community-associated Staphylococcus aureus ST121 clone causing fatal diseases in China and other CA-MRSA clones
Source: Virulence. 2023 Aug 3;14(1):2242547. doi: 10.1080/21505594.2023.2242547 (PMC10402838; doi:10.1080/21505594.2023.2242547)
Supplement: Supplemental Material [file KVIR_A_2242547_SM5961.zip › Table S2.docx]

**Table S2. Brief clinical records of CA-SA infections caused by ST121 isolates.**

| **Strains ID/Case** | **Separation time** | **Region** | **Age** | **Sex** | **Clinical diagnosis** | **Outcome** |
| --- | --- | --- | --- | --- | --- | --- |
| SKLX001130 | 2011 | Guangdong | 33 | Male | Skin abscess | Cured |
| SKLX001201 | 2011 | Guangdong | 28 | Female | Skin abscess | Cured |
| SKLX002403 | 2011 | Xinjiang | 41 | Female | Folliculitis | Cured |
| SKLX002405 | 2011 | Xinjiang | 37 | Male | Cellulitis | Cured |
| SKLX002565 | 2011 | Xinjiang | 61 | Female | Pneumonia | Death |
| SKLX002578 | 2011 | Xinjiang | 17 | Male | Furuncle | Cured |
| SKLX51514 | 2014 | Jiangsu | 6 | Female | SSSS | Cured |
| SKLX53305 | 2015 | Zhejiang | 5 | Female | Skin abscess | Death |
| SKLX54055 | 2015 | Anhui | 35 | Male | Impetigo | Cured |
| SKLX55935 | 2016 | Shandong | 54 | Female | Skin abscess | Death |
| SKLX56593 | 2016 | Fujian | 2 weeks | Male | SSSS | Death |
| SKLX61207 | 2017 | Henan | 43 | Female | Impetigo | Cured |
| SKLX61451 | 2017 | Xinjiang | 59 | Female | Pneumonia | Cured |
| SKLX61461 | 2017 | Xinjiang | 72 | Male | Pneumonia | Cured |
| SKLX61473 | 2017 | Xinjiang | 63 | Female | Pneumonia | Cured |
| SKLX61474 | 2017 | Xinjiang | 55 | Male | Pneumonia | Cured |
| SKLX61475 | 2017 | Xinjiang | 61 | Female | Pneumonia | Cured |
| SKLX61476 | 2017 | Xinjiang | 45 | Female | Pneumonia | Cured |
| SKLX61697 | 2017 | Henan | 35 | Male | Impetigo | Cured |
| SKLX63483 | 2018 | Anhui | 2 | Female | SSSS | Cured |
| SKLX63558 | 2018 | Anhui | 3 | Male | SSSS | Cured |
| SKLX79174 | 2018 | Hunan | 5 | Female | SSSS | Cured |
| SKLX108304 | 2019 | Jiangxi | 3 weeks | Male | SSSS | Cured |
| SKLX115129 | 2019 | Jiangxi | 2 weeks | Female | SSSS | Cured |
| SKLX115138 | 2019 | Jiangxi | 2 weeks | Male | SSSS | Cured |
| SKLX105761 | 2019 | Gansu | 4 months | Female | Pneumonia | Death |
| SKLX113906 | 2019 | Hubei | 66 | Male | Pneumonia | Death |
| SKLX88925 | 2019 | Hunan | 23 | Female | Impetigo | Cured |

**SSSS, staphylococcal scalded skin syndrome.**
